# Neglected knee swelling: A case report of massive synovial chondromatosis

**DOI:** 10.1016/j.ijscr.2024.110636

**Published:** 2024-11-21

**Authors:** Faten Limaiem, Mohamed Amine Gharbi, Ramzi Bouzidi

**Affiliations:** a University of Tunis El Manar, Faculty of Medicine of Tunis, 1007, Tunisia; b Pathology Department, Hospital Mongi Slim, La Marsa, Tunisia; c Department of Orthopedic Surgery, Hospital Mongi Slim, La Marsa, Tunisia

**Keywords:** Synovial chondromatosis, Knee joint, Surgery, Arthrotomy, Pathology

## Abstract

**Introduction and importance:**

Synovial chondromatosis (SC) is a rare condition characterized by the formation of cartilaginous loose bodies within joints, primarily arising from synovial membrane metaplasia. Misdiagnosis often occurs due to nonspecific symptoms, potentially leading to joint damage if inadequately managed.

**Case presentation:**

A 68-year-old man, presented with persistent knee pain, swelling, and limited mobility. Imaging revealed a sizable calcific mass within the knee joint, prompting surgical intervention. Arthrotomy was performed to excise an 18 cm × 15 cm × 6.5 cm mass containing multiple fused hyaline cartilaginous nodules circumscribed by a thick rim of adipose tissue. Histological examination confirmed a diagnosis of SC. Following surgery, the patient underwent physical therapy and experienced full recovery without any recurrence.

**Clinical discussion:**

This case underscores the challenges in managing neglected advanced SC, emphasizing the role of timely surgical intervention and postoperative care in achieving favorable outcomes. The definitive diagnosis of SC relies on histopathological examination.

**Conclusions:**

SC, an infrequent cause of knee swelling, requires early identification to prevent joint degeneration. Although malignant transformation to synovial chondrosarcoma is rare, a vigilant approach is crucial. Recurrences or resistant cases should raise suspicion for malignant transformation.

## Highlights

• Synovial chondromatosis is an uncommon cause of knee swelling and its clinical presentation is not specific.
• Prompt identification of synovial chondromatosis is crucial to prevent joint damage.
• Possible complications may involve secondary osteoarthritis, malignant transformation, and recurrence.
• Close monitoring is key to early detection of potential malignancy in recurrent or treatment-resistant cases.
• Our case is remarkable due to the large, neglected 18 cm mass causing knee swelling for five years.

## 1 Introduction

Synovial chondromatosis (SC), also referred to as synovial osteochondromatosis, is a rare and benign condition characterized by the formation of intra-articular cartilaginous loose bodies due to synovial membrane metaplasia [[1], [2], [3]]. While typically a primary condition, SC can also emerge secondarily from underlying joint pathologies or trauma [1]. Although the knee joint is frequently affected, SC can also manifest in the hip, elbow, shoulder, and ankle [2]. The lack of specific early symptoms often leads to the misdiagnosis of knee SC as meniscal issues or osteoarthritis [1,2]. Despite its generally non-aggressive and self-limiting course, inadequate management of SC can lead to substantial joint damage. Surgical intervention, the mainstay of treatment for SC, focuses on removing loose bodies and addressing synovial issues to prevent recurrence [4]. This paper presents a case report of a neglected giant SC affecting the knee joint. By elucidating this case, we aim to address the challenges and considerations in managing advanced SC cases, emphasizing the necessity of timely and appropriate interventions to safeguard joint health and function.

This case report adheres to the SCARE Criteria [5].

## 2 Case presentation

### 2.1 Patient history and presenting complaint

A 68-year-old Tunisian man with a history of hypertension, type 2 diabetes managed with oral antidiabetic drugs, and a background of coronary artery disease with stent placement, presented with complaints of swelling, pain, and limited mobility in his left knee joint persisting for five years. He denied any history of trauma. The patient initially consulted a private practice physician for pain relief and diagnostic tests. Despite initial treatment with nonsteroidal anti-inflammatory drugs and rest, the pain persisted, leading to a decline in knee mobility. As knee swelling worsened, significantly impacting daily activities, the patient sought further care at our hospital.

### 2.2 Physical examination findings

Upon examination, the left knee of the patient displayed restricted movement, with flexion limited to 90 degrees and extension to 20 degrees.

### 2.3 Diagnostic workup

**Fig. 1:**
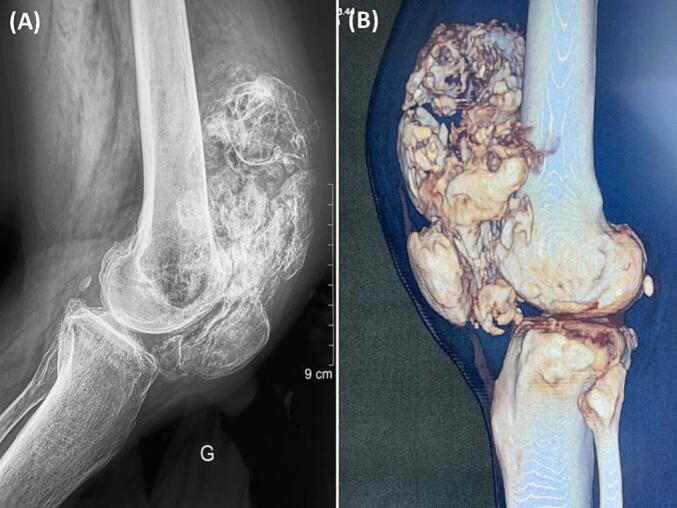
A: Lateral plain X-ray of the left knee revealing an irregular calcific mass extending from below the patella to the suprapatellar region. B: 3D CT scan sagittal view showing multiple loose bodies in the patellofemoral and tibiofemoral joints.

X-rays of the left knee and femur revealed degenerative alterations and a sizable, irregular calcific mass extending from below the patella to the suprapatellar area (Fig. 1A). The CT scan of the left knee and femur revealed multiple popcorn-like calcifications beneath the quadriceps and within the Hoffa fat pad, along with a moderate amount of intra-articular fluid effusion (Fig. 1B), suggestive of SC. Additionally, it indicated the presence of bicompartmental osteoarthritis. The MRI of the left knee and femur showed an enlarged suprapatellar recess with multiple focal lesions exhibiting an osteo-cartilaginous signal. At the center of these lesions, the signal matched that of the bone marrow, surrounded by a hyperintense peripheral ring displaying cartilage-like signals on T2-weighted and T2 FATSAT sequences. Furthermore, there was degenerative alteration of the patella and edematous changes in the femoral condyles and tibial plateau. A pre-operative surgical biopsy of the lesion confirmed the diagnosis of SC through histopathological examination.

### 2.4 Surgery

**Fig. 2:**
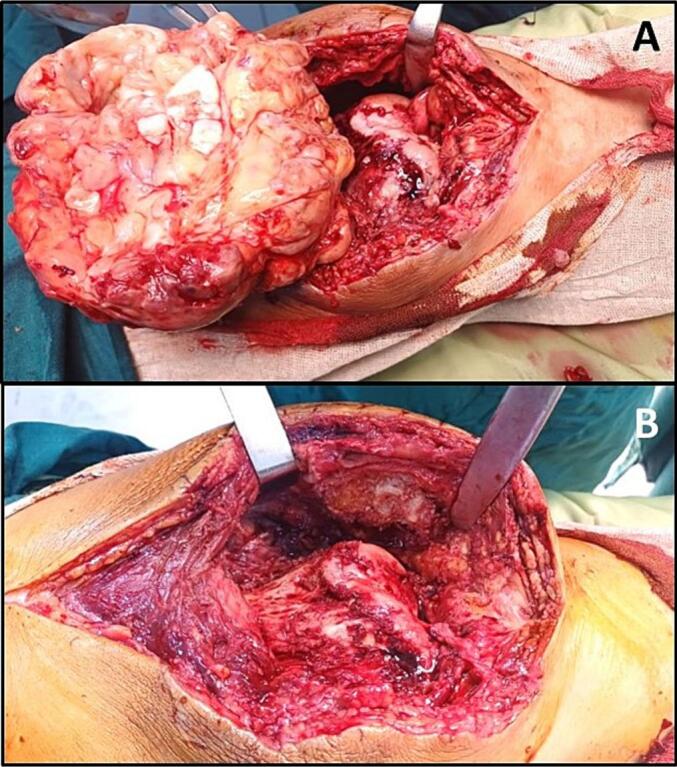
A: Intraoperative image depicting the excision of a large cartilaginous mass from the left knee. B: Intraoperative image of the left knee after removal of the mass.

After careful consideration, surgical intervention was deemed necessary and the patient consented. We opted for open surgery to completely remove the large mass, as the osteochondromas were clustered into a single block beneath the quadriceps, making arthroscopic removal impractical. Under spinal anesthesia, an anterior longitudinal incision provided access to the knee joint. The mass was meticulously exposed, freed from surrounding tissues, and excised (Fig. 2A and B). Additionally, a synovectomy was performed to eliminate synovial tissue. The surgical site was thoroughly irrigated, and the excised mass was sent for pathology evaluation. Intraoperatively, the cartilage of the left knee showed sufficient preservation, confirmed by X-ray findings of moderate arthrosis, thus negating the necessity for total knee arthroplasty in this patient.

### 2.5 Pathology findings

**Fig. 3:**
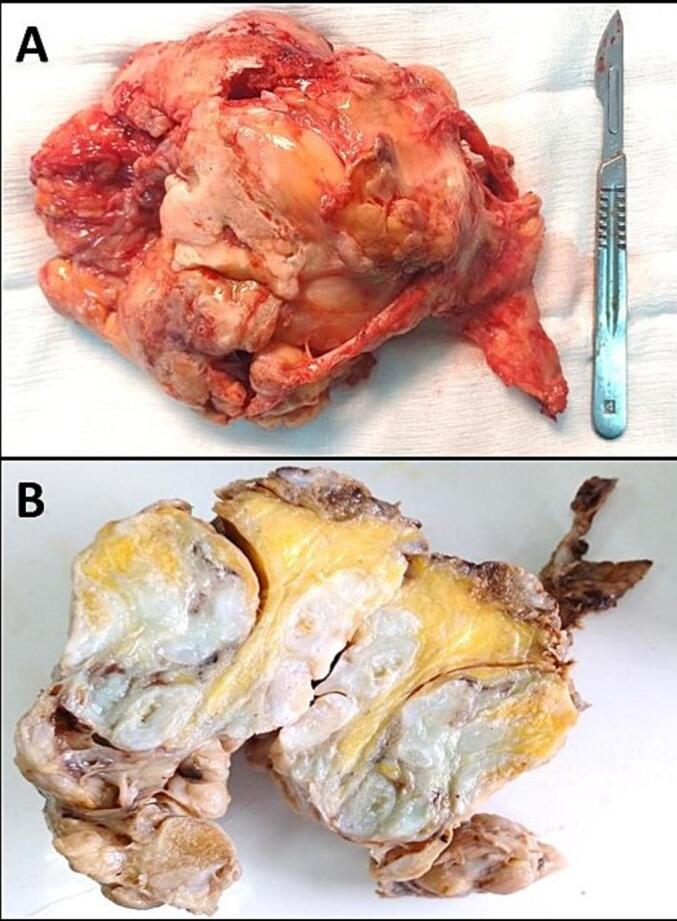
A: Macroscopic examination of the excised mass post-surgical removal. B: Macroscopic examination of the excised mass after formalin fixation. On cut section, there were multiple cartilaginous nodules of variable size circumscribed by a thick rim of adipose tissue (lipomatous villous proliferation of the synovium).

**Fig. 4:**
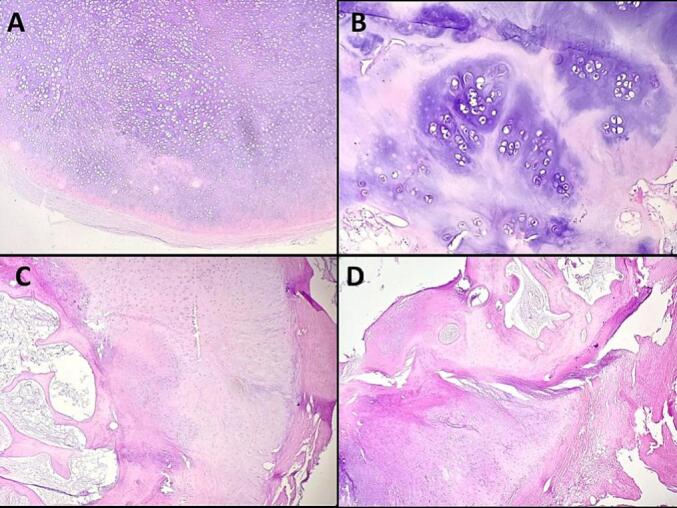
A: Mature hyaline cartilage beneath synovial layer. (Hematoxylin and eosin, magnification ×100). B: High-power magnification revealing mature hyaline cartilage showing clustering of chondrocytes (Hematoxylin and eosin, magnification ×400). C + D : Enchondral ossification. (Hematoxylin and eosin, magnification ×100).

The excised mass (Fig. 3A) weighed 577 g and measured 18 cm × 15 cm × 6.5 cm, exhibiting a hard consistency. Upon sectioning, it showcased numerous grayish-white, smooth, coalescing nodules of varying sizes, enveloped by a dense rim of adipose tissue (Fig. 3B). Histological examination, (Fig. 4A and B), revealed multiple mature hyaline cartilaginous nodules with chondrocyte clustering, minimal to moderate atypia, and increased cellularity. Notably, there was no infiltration into adjacent bone, but endochondral ossification was evident (Fig. 4C and D). The confirmed pathological diagnosis was SC.

### 2.6 Postoperative course and follow-up

**Fig. 5:**
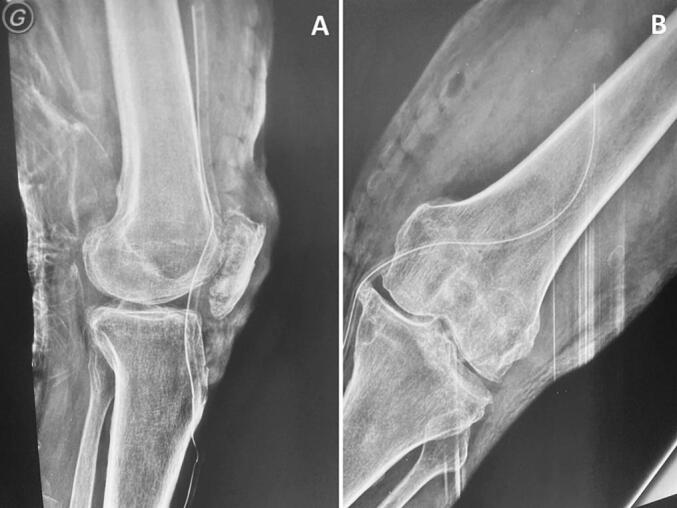
(A + B) Postoperative radiographs of the left knee joint showing the successful excision of the mass with moderate Ahlback stage 2 osteoarthritis.

Postoperative radiographs of the left knee revealed successful mass removal along with moderate Ahlback stage 2 osteoarthritis (Fig. 5). The recovery phase proceeded smoothly. The patient engaged in physical therapy sessions involving early active and passive exercises. Over the four-month follow-up period, the patient showed no signs of recurrence, fully restoring range of motion without encountering pain or complications during the latest check-up.

## 3 Discussion

SC is a rare condition, more prevalent in males aged 30 to 50, with an estimated incidence of 1 in 100,000 in the general adult population [6]. The exact cause is not fully understood, but it is thought to arise from the abnormal proliferation of undifferentiated mesenchymal stem cells within the synovium, leading to the development of cartilaginous nodules [2,4,7]. These nodules can detach and form loose bodies within the joint cavity or synovial folds, potentially undergoing calcification and ossification over time. SC typically affects a single joint but can rarely involve multiple joints [2,4]. It presents in primary and secondary forms and progresses through three stages: synovial metaplasia (stage 1), active inflammatory synovitis with early loose body formation (stage 2), and calcification of loose bodies with reduced synovial inflammation (stage 3) [2,4,7,8]. The clinical presentation of SC lacks specificity [2,4]. Patients may remain asymptomatic, but those with symptoms commonly complain of joint pain as the primary issue [2,4]. This pain is often accompanied by swelling, restricted range of motion, and intermittently, crepitus and mechanical locking [9]. The diagnosis of SC involves utilizing various imaging modalities such as plain radiographs, CT scans, or MRI, with MRI being the preferred method due to its superior soft tissue contrast capabilities [10]. Imaging studies demonstrate calcifications within and surrounding the knee joint, with intra-articular lesions prevalent in 70 % to 95 % of cases and extra-articular loose bodies in 20 % to 50 % of cases [10]. However, approximately 30 % of cases may initially evade detection due to the absence of calcifications in nodules [10]. Unique calcification patterns like ‘dot-and-comma,’ ‘ring-and-arc,’ or ‘popcorn-like’ structures are indicative of a chondroid origin [10]. Disease progression leads to arthritic changes, resulting in joint space obliteration and immobility. MRI of the knee shows hypointense **signal** and signal alterations ranging from low to high intensity in T1W and T2W sequences [10]. Contrast-enhanced MRI assists in differentiating pathologies based on synovial changes, calcification patterns, mineralization, and bony erosions [10]. The management of SC commonly involves surgical interventions [2,4]. Various approaches, including minimally invasive arthroscopic techniques and open surgical procedures that entail partial or subtotal synovectomy, are employed for treatment [11]. In our case, an open surgical approach was necessary for the complete removal of this large mass. This decision was particularly important because the osteochondromas had clustered into a single large block, primarily located at the cul-de-sac under the quadriceps, making arthroscopic excision impossible. Studies comparing the outcomes of synovectomy combined with the removal of loose bodies have demonstrated superior results in comparison to the removal of loose bodies alone [12]. In cases where osteoarthritis coexists with SC and depending on the extent of joint damage, total knee arthroplasty may be necessary [13]. In the case of our patient, during the surgery, the cartilage in the left knee was found to be adequately preserved, as supported by X-ray results indicating moderate arthrosis. Consequently, the need for total knee arthroplasty was ruled out for this patient. After surgery, the management plan involves gradually improving the range of motion and strengthening the muscle groups around the joint [1,11]. Macroscopically, SC appears as numerous grayish-white nodules that can be smooth or irregular, ranging in size and found either as loose bodies within the joint or connected to the synovium [1,14]. Histological analysis of SC demonstrates the presence of numerous hyaline cartilaginous nodules within the synovium or freely within joint spaces, accompanied by chondrocyte clustering, minimal atypia, and heightened cellularity, with the potential for calcification or endochondral ossification in longstanding instances [1,11,14]. The differential diagnoses of SC include crystal deposition diseases, osteocartilaginous loose bodies, osteochondritis dissecans, synovial hemangioma, lipoma arborescent, pigmented villonodular synovitis, tenosynovial giant cell tumor, tumoral calcinosis, and peri-articular melorheostosis [11]. Evaluation of potential malignant lesions such as low-grade chondrosarcoma and synovial sarcoma is crucial in this comprehensive differential diagnosis approach, with MRI findings playing a key role in accurate differentiation [1,2,11].

In summary, our case is distinguished by the substantial size of the mass that led to knee swelling, neglected by the patient for five years. Furthermore, the gross findings of SC in our case are remarkable, with multiple cartilaginous loose bodies merging within a thick rim of adipose tissue, forming a large mass. The diagnosis was initially made through imaging studies and subsequently confirmed by histopathological examination. SC is an infrequent cause of knee swelling, necessitating timely identification and intervention to prevent joint degeneration. While malignant transformation to synovial chondrosarcoma is rare, a vigilant approach is crucial for detection [1,4,5,11]. Recurrence or resistance should prompt consideration of malignant transformation [15].

## Author contribution

**Dr. Faten LIMAIEM**: Prepared, organized, wrote, and edited all aspects of the manuscript.

**Dr. Mohamed Amine GHARBI,** and **Pr. Ramzi BOUZIDI**: Read, edited, and approved the final version of the manuscript. Contributed to data acquisition, analysis, and interpretation. Provided final approval of the manuscript before its submission.

## Consent

Written informed consent was obtained from the patient for publication of this case report and accompanying images. A copy of the written consent is available for review by the Editor-in-Chief of this journal on request.

## Ethical approval

Ethical approval for this study was provided by the Ethical Committee of Mongi Slim University Hospital, Marsa, Tunisia.

## Guarantor

Dr. Faten LIMAIEM.

## Provenance and peer review

Not commissioned, externally peer-reviewed.

## Funding

This research did not receive any specific grant from funding agencies in the public, commercial, or not-for-profit sectors.

## Conflict of interest statement

None declared.
